# Heptaaqua­(3,4,5,6-tetra­chloro­phthalato-κ*O* ^1^)erbium(III) 2-carb­oxy-3,4,5,6-tetra­chloro­benzoate–3,4,5,6-tetra­chloro­phthalic acid–water (1/1/1)

**DOI:** 10.1107/S1600536812016923

**Published:** 2012-04-25

**Authors:** Yan Ouyang, Jia Shao, Lanfang Hao, Jixin Lu

**Affiliations:** a Tianjin Key Laboratory on Technologies Enabling Development of Clinical Therapeutics and Diagnostics (Theranostics), School of Pharmacy, Tianjin Medical University, Tianjin 300070, People’s Republic of China

## Abstract

In the three-dimensional tetra­chloro­phthalate-bridged title complex [Er(C_8_Cl_4_O_4_)(H_2_O)_7_](C_8_HCl_4_O_4_)·C_8_H_2_Cl_4_O_4_·H_2_O, the Er^III^ ion is coordinated in form of a distorted square antiprism by an O atom of a tetra­chloro­phthalate ligand and by seven water O atoms. Extensive hydrogen bonds establish a layered network structure extending parallel to (001).

## Related literature

For transition metal tetra­chloro­phthalato complexes, see: Ma *et al.* (2009 ▶). For lanthanide tetra­chloro­phthalato complexes, see: Liang *et al.* (2004 ▶); Xu *et al.* (2008 ▶).
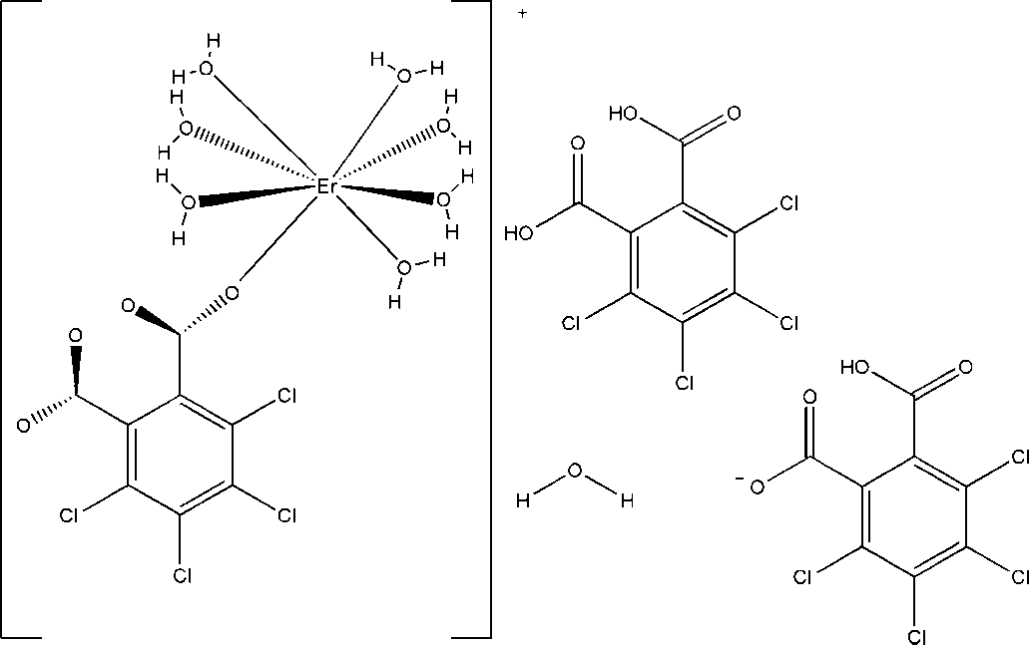


## Experimental

### 

#### Crystal data

[Er(C_8_Cl_4_O_4_)(H_2_O)_7_](C_8_HCl_4_O_4_)·C_8_H_2_Cl_4_O_4_·H_2_O
*M* *_r_* = 1220.05
Triclinic, 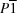

*a* = 6.865 (2) Å
*b* = 16.229 (5) Å
*c* = 19.019 (7) Å
α = 67.430 (8)°
β = 86.597 (13)°
γ = 81.626 (14)°
*V* = 1935.9 (11) Å^3^
*Z* = 2
Mo *K*α radiation
μ = 3.08 mm^−1^
*T* = 294 K
0.16 × 0.08 × 0.08 mm

#### Data collection

Rigaku Saturn diffractometer
Absorption correction: multi-scan (*CrystalClear*; Rigaku, 1999 ▶) *T*_min_ = 0.639, *T*_max_ = 0.791
14352 measured reflections
8732 independent reflections
7423 reflections with *I* > 2σ(*I*)
*R*_int_ = 0.023

#### Refinement

*R*[*F* ^2^ > 2σ(*F* ^2^)] = 0.024
*wR*(*F* ^2^) = 0.063
*S* = 1.00
8732 reflections
527 parameters
3 restraints
H atoms treated by a mixture of independent and constrained refinement
Δρ_max_ = 0.75 e Å^−3^
Δρ_min_ = −1.29 e Å^−3^

### 

Data collection: *CrystalClear* (Rigaku, 1999 ▶); cell refinement: *CrystalClear*; data reduction: *CrystalStructure* (Rigaku, 1999 ▶); program(s) used to solve structure: *SIR92* (Altomare *et al.*, 1994 ▶); program(s) used to refine structure: *SHELXL97* (Sheldrick, 2008 ▶); molecular graphics: *XP* (Sheldrick, 2008 ▶); software used to prepare material for publication: *CrystalStructure*.

## Supplementary Material

Crystal structure: contains datablock(s) global, I. DOI: 10.1107/S1600536812016923/bt5856sup1.cif

Structure factors: contains datablock(s) I. DOI: 10.1107/S1600536812016923/bt5856Isup2.hkl

Additional supplementary materials:  crystallographic information; 3D view; checkCIF report

## Figures and Tables

**Table 1:** Hydrogen-bond geometry (Å, °)

*D*—H⋯*A*	*D*—H	H⋯*A*	*D*⋯*A*	*D*—H⋯*A*
O7—H7⋯O6^i^	0.82 (2)	1.77 (2)	2.566 (3)	162 (3)
O10—H10⋯O4^ii^	0.82 (3)	1.77 (3)	2.566 (3)	164 (3)
O12—H12⋯O5^iii^	0.82 (3)	1.77 (2)	2.583 (3)	174 (5)
O13—H13*A*⋯O9^iv^	0.84	2.57	2.969 (3)	110
O13—H13*A*⋯O2^v^	0.84	2.03	2.814 (3)	155
O13—H13*B*⋯O20^vi^	0.84	1.93	2.733 (3)	159
O14—H14*A*⋯O2^v^	0.85	1.82	2.663 (3)	169
O14—H14*B*⋯O3^vii^	0.85	1.92	2.724 (3)	157
O15—H15*A*⋯O3	0.84	2.03	2.868 (3)	169
O15—H15*B*⋯O4^viii^	0.85	1.90	2.738 (3)	168
O16—H16*A*⋯O8^ix^	0.85	1.95	2.774 (3)	166
O16—H16*B*⋯O3^vii^	0.85	1.98	2.764 (3)	153
O17—H17*A*⋯O6^ix^	0.84	1.92	2.750 (3)	168
O17—H17*B*⋯Cl5^ii^	0.84	2.83	3.614 (3)	157
O18—H18*A*⋯O20^vi^	0.84	1.93	2.741 (4)	161
O18—H18*B*⋯O5^x^	0.84	2.30	2.809 (4)	119
O19—H19*A*⋯O11^iv^	0.84	2.09	2.909 (3)	167
O19—H19*B*⋯O5^xi^	0.84	2.51	3.119 (3)	131
O19—H19*B*⋯O8^ix^	0.84	2.31	3.010 (3)	141
O20—H20*A*⋯O11^vii^	0.85	2.04	2.781 (3)	146
O20—H20*B*⋯O10	0.85	2.17	2.834 (3)	134
O20—H20*B*⋯O12	0.85	2.59	3.080 (3)	118

Symmetry codes: (i) 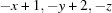; (ii) 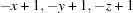; (iii) 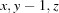; (iv) 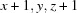; (v) 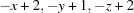; (vi) 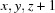; (vii) 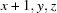; (viii) 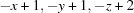; (ix) 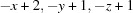; (x) 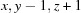; (xi) 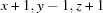.

**Fig. 1.**
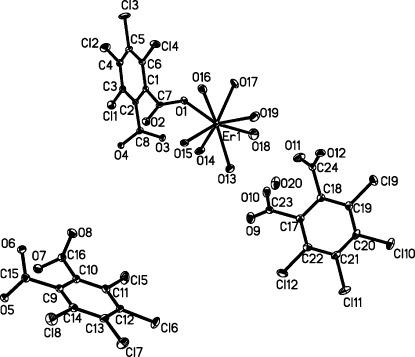
The molecular structure of the title compound, with displacement ellipsoids drawn at the 30% probability level. [Symmetry codes:(i) -x, -y+1, -z; (ii) x+1, y, z; (iii) -x-1, -y+1, -z; (iv) -x+1, -y+1, -z; (v) x-1, y-1, z; (vi) x, y-1, z; (vii) x-1, y, z; (viii) -x+1, -y+2, -z]. H atoms have been omitted for clarity.

**Fig. 2.**
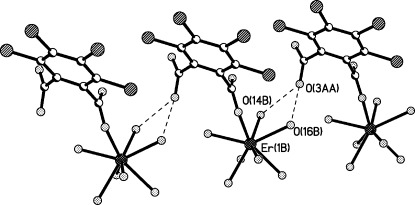
Fragment of the crystal structure of title complex showing the dimeric unit formed by hydrogen bonds with view along the a axis.

**Fig. 3.**
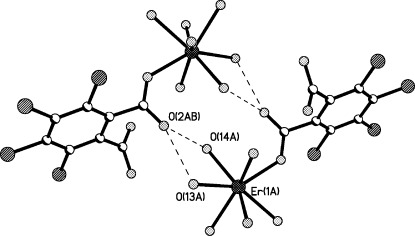
Fragment of the crystal structure of title complex showing the dimeric unit formed by hydrogen bonds with view along the b axis.

**Fig. 4.**
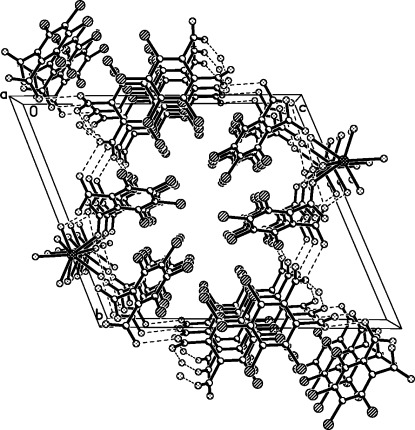
Packing diagram of the title compound.

## supplementary crystallographic information

### Comment

As a versatile bridge ligand, tcph_2_ anion (H_2_tcph=tetrachlorophthalic acid) with versatility of coordination modes was successfully used as bridge for the design and synthesis of a wide variety of polynuclear species, often having both interesting structures and properties. To date, most of the published work concerns transition metal tetrachlorophthalato complexes (Ma *et al.*, 2009). In order to provide more examples of lanthanide–tetrachlorophthalate complexes with novel structure in this study, we selected Erbium(III) ion to obtain the title complex.

Single crystal X-ray diffraction analysis reveals that complex (I) consists of a [Er(tcph)(H_2_O)_7_]^+^ cation(Figure 1), a neutral (H_2_tcph) molecule, an (Htcph)^-^ anion and an uncoordinated water molecule. Selected bond lengths and angles are presented in Table 1. The Er^III^ ion is coordinated by eight O atoms, one from a tcph ligand and others from coordinated water molecular. The Er–O band distances range from 2.291 (2) Å to 2.382 (2) Å. It is interesting that the complex contains several kinds of hydrogen bonds. The oxygen atoms from carboxylate act as acceptors and the coordinated water molecules as donors. Thus along *a* axis neighbouring mononuclear structural units form an unusual dimer by means of two short hydrogen bonds (O2–O13=2.814 (3) Å, O2–O14 = 2.663 (3) Å) between uncoordinated carboxylate O atom and coordinated water molecule (Figure 2), and Er–Er distance is 6.218 Å. Along *b* axis,two adjacent [Er(tcph)(H_2_O)_7_]^+^ cation are linked by short hydrogen bonds (O3–O16 = 2.732 (3) Å, O3–O14 = 2.764 (3) Å) (Figure 3), and Er–Er distance is 6.865 Å. Furthermore, there are some hydrogen bonds between [Er(tcph)(H_2_O)_7_]^+^ cations and (Htcph)^-^ anions. A two-dimensional network is constructed *via* a series of extensive hydrogen bonds. (Figure. 4) There are many hydrogen bonds owing to the presence of fully deprotonated carboxylate groups and a significant number of water molecules. Hydrogen bonding distances and angles are presented in Table 2. For clarity the conventional description of hydrogen bonding structural parameters has been adopted: D–A indicates the distance between a donor D atom and acceptor A atom, H–A the distance between a donor hydrogen atom bound to D and acceptor, while DHA indicates the angle.

IR spectra of the title complex exhibit the bands expected for the carbonyl stretching (1721 cm_-1_), the bands for water stretching (3200–3100 cm_-1_) and the bands for benzene (1500–1410 cm_-1_). The absorption bands in the spectrum of the title complex were red-shifted relative to strong hydrogen bonds.

### Experimental

A solution of Er(NO_3_)_3_.6H_2_O(0.5 mmol) in H_2_O (10 ml) was added to a suspension of a suspension of H_2_tcph (0.5 mmol) in H_2_O (30 ml). The mixture was stirred at room temperature for 30 min. After filtration,the solution was left undisturbed and white crystal was obtain after 15 days.analysis, calculated for C_12_H_8_Cl_12_ErO_20_: C 23.63, H 1.57%; found: C 23.55, H 1.50%.

### Refinement

The three hydroxyl hydrogen atoms were refined isotropically with distance restraints of O–H = 0.82 (1)Å. All others were refined using a riding model.

### Crystal data

**Table:**

[Er(C_8_Cl_4_O_4_)(H_2_O)_7_](C_8_HCl_4_O_4_)·C_8_H_2_Cl_4_O_4_·H_2_O	*Z* = 2
*M**_r_* = 1220.05	*F*(000) = 1190
Triclinic, *P*1	*D*_x_ = 2.093 Mg m^−^^3^
*a* = 6.865 (2) Å	Mo *K*α radiation, λ = 0.71070 Å
*b* = 16.229 (5) Å	Cell parameters from 6109 reflections
*c* = 19.019 (7) Å	θ = 2.2–28.0°
α = 67.430 (8)°	µ = 3.08 mm^−^^1^
β = 86.597 (13)°	*T* = 294 K
γ = 81.626 (14)°	Block, colorless
*V* = 1935.9 (11) Å^3^	0.16 × 0.08 × 0.08 mm

### Data collection

**Table:**

Rigaku Saturn diffractometer	8732 independent reflections
Radiation source: fine-focus sealed tube	7423 reflections with *I* > 2σ(*I*)
Graphite monochromator	*R*_int_ = 0.023
Detector resolution: 7.31 pixels mm^-1^	θ_max_ = 27.6°, θ_min_ = 3.1°
ω scans	*h* = −8→8
Absorption correction: multi-scan (*CrystalClear*; Rigaku, 1999)	*k* = −21→19
*T*_min_ = 0.639, *T*_max_ = 0.791	*l* = −24→22
14352 measured reflections	

### Refinement

**Table:**

Refinement on *F*^2^	Primary atom site location: structure-invariant direct methods
Least-squares matrix: full	Secondary atom site location: difference Fourier map
*R*[*F*^2^ > 2σ(*F*^2^)] = 0.024	Hydrogen site location: inferred from neighbouring sites
*wR*(*F*^2^) = 0.063	H atoms treated by a mixture of independent and constrained refinement
*S* = 1.00	*w* = 1/[σ^2^(*F*_o_^2^) + (0.0349*P*)^2^] where *P* = (*F*_o_^2^ + 2*F*_c_^2^)/3
8732 reflections	(Δ/σ)_max_ = 0.003
527 parameters	Δρ_max_ = 0.75 e Å^−^^3^
3 restraints	Δρ_min_ = −1.29 e Å^−^^3^

### Special details

**Table:**

Geometry. All e.s.d.'s (except the e.s.d. in the dihedral angle between two l.s. planes) are estimated using the full covariance matrix. The cell e.s.d.'s are taken into account individually in the estimation of e.s.d.'s in distances, angles and torsion angles; correlations between e.s.d.'s in cell parameters are only used when they are defined by crystal symmetry. An approximate (isotropic) treatment of cell e.s.d.'s is used for estimating e.s.d.'s involving l.s. planes.
Refinement. Refinement of *F*^2^ against ALL reflections. The weighted *R*-factor *wR* and goodness of fit *S* are based on *F*^2^, conventional *R*-factors *R* are based on *F*, with *F* set to zero for negative *F*^2^. The threshold expression of *F*^2^ > σ(*F*^2^) is used only for calculating *R*-factors(gt) *etc*. and is not relevant to the choice of reflections for refinement. *R*-factors based on *F*^2^ are statistically about twice as large as those based on *F*, and *R*- factors based on ALL data will be even larger.

### Fractional atomic coordinates and isotropic or equivalent isotropic displacement parameters (Å^2^)

**Table:**

	*x*	*y*	*z*	*U*_iso_*/*U*_eq_	
Er1	1.052036 (16)	0.306469 (7)	0.999997 (6)	0.01887 (4)	
Cl1	0.23002 (10)	0.64640 (5)	0.70586 (4)	0.03329 (16)	
Cl2	0.40662 (13)	0.62105 (6)	0.55990 (4)	0.0467 (2)	
Cl3	0.83345 (14)	0.52578 (6)	0.56071 (4)	0.0494 (2)	
Cl4	1.07603 (11)	0.44511 (5)	0.71106 (4)	0.03644 (17)	
Cl5	0.29591 (13)	0.70136 (5)	0.22781 (5)	0.0445 (2)	
Cl6	0.53509 (14)	0.63458 (5)	0.37689 (4)	0.0446 (2)	
Cl7	0.89471 (14)	0.72812 (6)	0.38416 (5)	0.0478 (2)	
Cl8	0.98710 (11)	0.90094 (5)	0.24659 (5)	0.03817 (18)	
Cl9	0.35448 (12)	−0.13570 (5)	0.39175 (5)	0.03755 (17)	
Cl10	0.25335 (11)	−0.14158 (5)	0.55549 (4)	0.03720 (18)	
Cl11	0.18423 (11)	0.03707 (6)	0.58473 (4)	0.03692 (17)	
Cl12	0.21450 (12)	0.21921 (5)	0.45154 (5)	0.03884 (18)	
O1	0.9205 (3)	0.39196 (12)	0.88147 (11)	0.0284 (4)	
O2	0.9131 (3)	0.53332 (13)	0.87227 (11)	0.0319 (5)	
O3	0.4634 (3)	0.49189 (12)	0.90040 (11)	0.0273 (4)	
O4	0.4521 (3)	0.64077 (12)	0.84992 (11)	0.0259 (4)	
O5	0.6653 (3)	1.03878 (12)	0.12137 (11)	0.0283 (4)	
O6	0.7917 (3)	0.95674 (12)	0.05412 (10)	0.0275 (4)	
O7	0.3216 (3)	0.95054 (14)	0.08330 (12)	0.0338 (5)	
O8	0.4082 (3)	0.82485 (14)	0.06107 (12)	0.0346 (5)	
O9	0.1761 (3)	0.27388 (14)	0.27974 (13)	0.0385 (5)	
O10	0.4774 (3)	0.21429 (13)	0.26018 (12)	0.0334 (5)	
O11	0.2508 (3)	0.07482 (16)	0.22111 (12)	0.0402 (5)	
O12	0.5607 (3)	0.02273 (14)	0.25825 (12)	0.0294 (4)	
O13	1.0399 (3)	0.29762 (13)	1.12704 (11)	0.0337 (5)	
H13A	1.0773	0.3381	1.1385	0.051*	
H13B	0.9765	0.2627	1.1627	0.051*	
O14	1.1809 (3)	0.42624 (13)	1.00583 (11)	0.0277 (4)	
H14A	1.1573	0.4452	1.0417	0.042*	
H14B	1.2490	0.4605	0.9712	0.042*	
O15	0.7405 (3)	0.37720 (15)	1.01578 (11)	0.0361 (5)	
H15A	0.6606	0.4053	0.9796	0.054*	
H15B	0.6831	0.3636	1.0587	0.054*	
O16	1.3350 (3)	0.33085 (13)	0.91965 (13)	0.0357 (5)	
H16A	1.4251	0.2865	0.9297	0.054*	
H16B	1.3832	0.3802	0.8994	0.054*	
O17	1.0434 (3)	0.21430 (13)	0.93003 (12)	0.0358 (5)	
H17A	1.0812	0.1592	0.9399	0.054*	
H17B	0.9955	0.2413	0.8862	0.054*	
O18	0.8761 (4)	0.18711 (16)	1.07183 (15)	0.0521 (7)	
H18A	0.8605	0.1666	1.1193	0.078*	
H18B	0.8347	0.1562	1.0506	0.078*	
O19	1.3023 (3)	0.18593 (16)	1.06132 (12)	0.0436 (6)	
H19A	1.2811	0.1620	1.1082	0.065*	
H19B	1.4033	0.1611	1.0469	0.065*	
O20	0.8655 (3)	0.15500 (16)	0.22431 (14)	0.0484 (7)	
H20A	0.9651	0.1161	0.2414	0.073*	
H20B	0.7703	0.1443	0.2557	0.073*	
C1	0.7625 (4)	0.51060 (16)	0.77524 (14)	0.0185 (5)	
C2	0.5750 (4)	0.55643 (16)	0.77357 (14)	0.0191 (5)	
C3	0.4646 (4)	0.59134 (17)	0.70701 (15)	0.0220 (5)	
C4	0.5435 (4)	0.58012 (18)	0.64150 (15)	0.0268 (6)	
C5	0.7336 (4)	0.53640 (18)	0.64217 (15)	0.0272 (6)	
C6	0.8423 (4)	0.50157 (17)	0.70945 (15)	0.0221 (5)	
C7	0.8776 (4)	0.47622 (17)	0.84880 (14)	0.0206 (5)	
C8	0.4899 (3)	0.56417 (17)	0.84657 (14)	0.0197 (5)	
C9	0.6767 (4)	0.88031 (17)	0.17843 (15)	0.0214 (5)	
C10	0.5244 (4)	0.83474 (17)	0.17318 (15)	0.0222 (5)	
C11	0.4843 (4)	0.75752 (18)	0.23404 (16)	0.0254 (6)	
C12	0.5953 (4)	0.72585 (18)	0.30030 (15)	0.0273 (6)	
C13	0.7518 (4)	0.76896 (18)	0.30458 (15)	0.0275 (6)	
C14	0.7928 (4)	0.84635 (18)	0.24325 (16)	0.0247 (6)	
C15	0.7153 (4)	0.96574 (17)	0.11282 (15)	0.0213 (5)	
C16	0.4111 (4)	0.86923 (17)	0.09957 (15)	0.0230 (5)	
C17	0.2958 (3)	0.12420 (17)	0.36129 (15)	0.0206 (5)	
C18	0.3315 (4)	0.04388 (17)	0.34871 (15)	0.0208 (5)	
C19	0.3178 (4)	−0.03821 (17)	0.40924 (16)	0.0226 (5)	
C20	0.2706 (4)	−0.04027 (18)	0.48194 (15)	0.0251 (6)	
C21	0.2376 (3)	0.03923 (19)	0.49491 (15)	0.0233 (6)	
C22	0.2496 (4)	0.12182 (18)	0.43436 (15)	0.0231 (5)	
C23	0.3072 (4)	0.21319 (17)	0.29615 (15)	0.0214 (5)	
C24	0.3774 (4)	0.04817 (17)	0.26904 (15)	0.0220 (5)	
H7	0.281 (5)	0.970 (2)	0.0392 (10)	0.056 (12)*	
H10	0.477 (5)	0.2633 (14)	0.2246 (16)	0.054 (12)*	
H12	0.602 (6)	0.028 (3)	0.2158 (11)	0.064 (13)*	

### Atomic displacement parameters (Å^2^)

**Table:**

	*U*^11^	*U*^22^	*U*^33^	*U*^12^	*U*^13^	*U*^23^
Er1	0.02195 (6)	0.01616 (6)	0.01900 (7)	−0.00219 (4)	0.00221 (4)	−0.00767 (5)
Cl1	0.0258 (3)	0.0352 (4)	0.0349 (4)	0.0050 (3)	−0.0031 (3)	−0.0117 (3)
Cl2	0.0585 (5)	0.0546 (5)	0.0215 (4)	0.0068 (4)	−0.0125 (3)	−0.0117 (4)
Cl3	0.0618 (5)	0.0639 (6)	0.0207 (4)	0.0050 (5)	0.0104 (3)	−0.0202 (4)
Cl4	0.0315 (4)	0.0410 (4)	0.0374 (4)	0.0057 (3)	0.0091 (3)	−0.0206 (3)
Cl5	0.0492 (5)	0.0335 (4)	0.0461 (5)	−0.0213 (4)	0.0003 (4)	−0.0043 (4)
Cl6	0.0657 (5)	0.0316 (4)	0.0233 (4)	−0.0068 (4)	0.0111 (4)	0.0027 (3)
Cl7	0.0676 (6)	0.0442 (5)	0.0249 (4)	0.0038 (4)	−0.0170 (4)	−0.0073 (3)
Cl8	0.0342 (4)	0.0396 (4)	0.0431 (5)	−0.0058 (3)	−0.0090 (3)	−0.0168 (4)
Cl9	0.0471 (4)	0.0217 (3)	0.0424 (5)	−0.0033 (3)	0.0030 (3)	−0.0115 (3)
Cl10	0.0322 (4)	0.0335 (4)	0.0297 (4)	−0.0056 (3)	0.0017 (3)	0.0060 (3)
Cl11	0.0315 (4)	0.0611 (5)	0.0181 (3)	−0.0096 (3)	0.0049 (3)	−0.0144 (3)
Cl12	0.0490 (4)	0.0393 (4)	0.0386 (4)	−0.0105 (3)	0.0108 (3)	−0.0261 (4)
O1	0.0385 (11)	0.0187 (9)	0.0240 (10)	0.0065 (8)	−0.0019 (8)	−0.0073 (8)
O2	0.0493 (13)	0.0248 (10)	0.0242 (11)	−0.0067 (9)	−0.0018 (9)	−0.0115 (8)
O3	0.0356 (11)	0.0177 (9)	0.0225 (10)	−0.0026 (8)	0.0109 (8)	−0.0031 (8)
O4	0.0355 (11)	0.0163 (9)	0.0230 (10)	0.0007 (8)	0.0115 (8)	−0.0073 (8)
O5	0.0431 (12)	0.0178 (9)	0.0250 (11)	−0.0041 (8)	0.0067 (8)	−0.0102 (8)
O6	0.0392 (11)	0.0220 (9)	0.0179 (10)	0.0026 (8)	0.0044 (8)	−0.0069 (8)
O7	0.0482 (13)	0.0250 (10)	0.0255 (12)	0.0076 (9)	−0.0093 (9)	−0.0097 (9)
O8	0.0465 (13)	0.0285 (11)	0.0317 (12)	0.0005 (9)	−0.0085 (9)	−0.0155 (9)
O9	0.0318 (11)	0.0285 (11)	0.0431 (14)	0.0070 (9)	0.0055 (9)	−0.0051 (10)
O10	0.0322 (11)	0.0217 (10)	0.0344 (12)	−0.0001 (9)	0.0149 (9)	−0.0010 (9)
O11	0.0315 (11)	0.0609 (15)	0.0253 (11)	0.0046 (10)	−0.0040 (9)	−0.0163 (11)
O12	0.0269 (10)	0.0374 (11)	0.0233 (11)	0.0021 (9)	0.0057 (8)	−0.0139 (9)
O13	0.0485 (13)	0.0310 (11)	0.0239 (11)	−0.0151 (9)	0.0054 (9)	−0.0104 (9)
O14	0.0362 (11)	0.0267 (10)	0.0255 (11)	−0.0137 (8)	0.0140 (8)	−0.0143 (8)
O15	0.0289 (11)	0.0483 (13)	0.0215 (11)	0.0092 (10)	0.0071 (8)	−0.0090 (10)
O16	0.0350 (11)	0.0275 (11)	0.0451 (14)	−0.0061 (9)	0.0171 (9)	−0.0162 (10)
O17	0.0520 (13)	0.0212 (10)	0.0372 (13)	0.0088 (9)	−0.0138 (10)	−0.0172 (9)
O18	0.0636 (16)	0.0413 (14)	0.0581 (17)	−0.0322 (12)	0.0180 (13)	−0.0201 (12)
O19	0.0376 (12)	0.0476 (13)	0.0284 (12)	0.0136 (11)	0.0048 (9)	−0.0030 (10)
O20	0.0298 (12)	0.0402 (13)	0.0556 (16)	−0.0034 (10)	0.0149 (10)	0.0010 (11)
C1	0.0238 (12)	0.0144 (11)	0.0166 (12)	−0.0031 (10)	0.0041 (9)	−0.0055 (10)
C2	0.0244 (13)	0.0159 (11)	0.0164 (12)	−0.0044 (10)	0.0062 (9)	−0.0059 (10)
C3	0.0228 (13)	0.0191 (12)	0.0214 (13)	0.0006 (10)	0.0000 (10)	−0.0059 (10)
C4	0.0394 (16)	0.0225 (13)	0.0148 (13)	−0.0008 (12)	−0.0022 (11)	−0.0040 (11)
C5	0.0407 (16)	0.0233 (13)	0.0155 (13)	−0.0035 (12)	0.0096 (11)	−0.0068 (11)
C6	0.0261 (13)	0.0195 (12)	0.0204 (13)	−0.0006 (10)	0.0068 (10)	−0.0091 (10)
C7	0.0210 (12)	0.0238 (13)	0.0147 (12)	−0.0001 (10)	0.0054 (9)	−0.0064 (10)
C8	0.0154 (11)	0.0227 (13)	0.0174 (13)	−0.0007 (10)	0.0042 (9)	−0.0051 (10)
C9	0.0274 (13)	0.0163 (12)	0.0196 (13)	0.0005 (10)	0.0024 (10)	−0.0073 (10)
C10	0.0278 (13)	0.0163 (12)	0.0211 (13)	−0.0012 (10)	0.0021 (10)	−0.0065 (10)
C11	0.0307 (14)	0.0198 (13)	0.0264 (15)	−0.0027 (11)	0.0051 (11)	−0.0105 (11)
C12	0.0436 (16)	0.0186 (13)	0.0148 (13)	0.0030 (12)	0.0074 (11)	−0.0046 (10)
C13	0.0384 (16)	0.0243 (14)	0.0159 (13)	0.0065 (12)	−0.0027 (11)	−0.0066 (11)
C14	0.0300 (14)	0.0215 (13)	0.0234 (14)	0.0006 (11)	−0.0004 (11)	−0.0109 (11)
C15	0.0214 (12)	0.0204 (13)	0.0224 (14)	−0.0009 (10)	−0.0018 (10)	−0.0088 (11)
C16	0.0244 (13)	0.0216 (13)	0.0224 (14)	−0.0025 (10)	0.0013 (10)	−0.0080 (11)
C17	0.0151 (11)	0.0247 (13)	0.0205 (13)	−0.0008 (10)	0.0016 (9)	−0.0077 (11)
C18	0.0151 (11)	0.0239 (13)	0.0210 (13)	−0.0011 (10)	0.0014 (9)	−0.0068 (11)
C19	0.0175 (12)	0.0213 (13)	0.0263 (14)	−0.0017 (10)	−0.0002 (10)	−0.0065 (11)
C20	0.0164 (12)	0.0289 (14)	0.0204 (13)	−0.0053 (11)	−0.0011 (9)	0.0022 (11)
C21	0.0139 (12)	0.0357 (15)	0.0168 (13)	−0.0028 (11)	0.0019 (9)	−0.0065 (11)
C22	0.0177 (12)	0.0299 (14)	0.0250 (14)	−0.0066 (11)	0.0051 (10)	−0.0134 (12)
C23	0.0225 (13)	0.0208 (13)	0.0216 (13)	−0.0035 (10)	0.0019 (10)	−0.0089 (11)
C24	0.0252 (13)	0.0184 (12)	0.0214 (14)	0.0001 (10)	0.0025 (10)	−0.0078 (10)

### Geometric parameters (Å, º)

**Table:**

Er1—O14	2.291 (2)	O14—H14B	0.8480
Er1—O1	2.302 (2)	O15—H15A	0.8442
Er1—O15	2.336 (2)	O15—H15B	0.8488
Er1—O18	2.352 (2)	O16—H16A	0.8465
Er1—O17	2.361 (2)	O16—H16B	0.8511
Er1—O13	2.362 (2)	O17—H17A	0.8441
Er1—O19	2.376 (2)	O17—H17B	0.8395
Er1—O16	2.382 (2)	O18—H18A	0.8404
Cl1—C3	1.721 (3)	O18—H18B	0.8400
Cl2—C4	1.713 (3)	O19—H19A	0.8382
Cl3—C5	1.716 (3)	O19—H19B	0.8353
Cl4—C6	1.724 (3)	O20—H20A	0.8455
Cl5—C11	1.721 (3)	O20—H20B	0.8489
Cl6—C12	1.714 (3)	C1—C2	1.385 (3)
Cl7—C13	1.706 (3)	C1—C6	1.387 (3)
Cl8—C14	1.722 (3)	C1—C7	1.515 (3)
Cl9—C19	1.719 (3)	C2—C3	1.391 (4)
Cl10—C20	1.716 (3)	C2—C8	1.517 (3)
Cl11—C21	1.714 (3)	C3—C4	1.394 (4)
Cl12—C22	1.714 (3)	C4—C5	1.390 (4)
O1—C7	1.263 (3)	C5—C6	1.396 (4)
O2—C7	1.230 (3)	C9—C14	1.388 (4)
O3—C8	1.255 (3)	C9—C10	1.395 (4)
O4—C8	1.258 (3)	C9—C15	1.513 (4)
O5—C15	1.254 (3)	C10—C11	1.391 (4)
O6—C15	1.255 (3)	C10—C16	1.510 (4)
O7—C16	1.299 (3)	C11—C12	1.391 (4)
O7—H7	0.826 (10)	C12—C13	1.387 (4)
O8—C16	1.211 (3)	C13—C14	1.399 (4)
O9—C23	1.190 (3)	C17—C22	1.394 (4)
O10—C23	1.318 (3)	C17—C18	1.398 (4)
O10—H10	0.822 (10)	C17—C23	1.508 (4)
O11—C24	1.206 (3)	C18—C19	1.398 (4)
O12—C24	1.296 (3)	C18—C24	1.506 (4)
O12—H12	0.818 (10)	C19—C20	1.390 (4)
O13—H13A	0.8434	C20—C21	1.389 (4)
O13—H13B	0.8441	C21—C22	1.402 (4)
O14—H14A	0.8482		
			
O14—Er1—O1	91.91 (7)	C5—C4—C3	120.1 (2)
O14—Er1—O15	87.97 (8)	C5—C4—Cl2	120.0 (2)
O1—Er1—O15	71.56 (7)	C3—C4—Cl2	119.9 (2)
O14—Er1—O18	142.46 (8)	C4—C5—C6	119.5 (2)
O1—Er1—O18	113.68 (9)	C4—C5—Cl3	120.1 (2)
O15—Er1—O18	75.73 (9)	C6—C5—Cl3	120.5 (2)
O14—Er1—O17	145.21 (8)	C1—C6—C5	120.6 (2)
O1—Er1—O17	70.35 (7)	C1—C6—Cl4	119.8 (2)
O15—Er1—O17	112.66 (8)	C5—C6—Cl4	119.6 (2)
O18—Er1—O17	71.88 (9)	O2—C7—O1	126.4 (2)
O14—Er1—O13	70.63 (7)	O2—C7—C1	116.4 (2)
O1—Er1—O13	141.86 (7)	O1—C7—C1	117.0 (2)
O15—Er1—O13	74.12 (7)	O3—C8—O4	124.4 (2)
O18—Er1—O13	72.43 (9)	O3—C8—C2	116.5 (2)
O17—Er1—O13	140.32 (7)	O4—C8—C2	119.1 (2)
O14—Er1—O19	101.22 (9)	C14—C9—C10	119.8 (2)
O1—Er1—O19	141.31 (7)	C14—C9—C15	120.7 (2)
O15—Er1—O19	144.22 (7)	C10—C9—C15	119.6 (2)
O18—Er1—O19	76.24 (9)	C11—C10—C9	119.9 (2)
O17—Er1—O19	78.65 (8)	C11—C10—C16	121.5 (2)
O13—Er1—O19	76.55 (8)	C9—C10—C16	118.5 (2)
O14—Er1—O16	71.54 (7)	C10—C11—C12	120.2 (3)
O1—Er1—O16	76.86 (8)	C10—C11—Cl5	120.0 (2)
O15—Er1—O16	141.55 (7)	C12—C11—Cl5	119.8 (2)
O18—Er1—O16	138.68 (8)	C13—C12—C11	120.0 (2)
O17—Er1—O16	75.30 (8)	C13—C12—Cl6	120.3 (2)
O13—Er1—O16	124.91 (8)	C11—C12—Cl6	119.6 (2)
O19—Er1—O16	73.26 (8)	C12—C13—C14	119.8 (3)
C7—O1—Er1	130.39 (18)	C12—C13—Cl7	120.4 (2)
C16—O7—H7	108 (3)	C14—C13—Cl7	119.8 (2)
C23—O10—H10	109 (3)	C9—C14—C13	120.2 (3)
C24—O12—H12	121 (3)	C9—C14—Cl8	118.9 (2)
Er1—O13—H13A	121.2	C13—C14—Cl8	120.9 (2)
Er1—O13—H13B	125.0	O5—C15—O6	126.0 (2)
H13A—O13—H13B	112.4	O5—C15—C9	117.4 (2)
Er1—O14—H14A	123.8	O6—C15—C9	116.6 (2)
Er1—O14—H14B	125.4	O8—C16—O7	125.3 (3)
H14A—O14—H14B	110.6	O8—C16—C10	122.5 (2)
Er1—O15—H15A	123.3	O7—C16—C10	112.2 (2)
Er1—O15—H15B	122.4	C22—C17—C18	119.8 (2)
H15A—O15—H15B	111.6	C22—C17—C23	120.0 (2)
Er1—O16—H16A	115.7	C18—C17—C23	120.2 (2)
Er1—O16—H16B	126.0	C19—C18—C17	119.8 (2)
H16A—O16—H16B	110.6	C19—C18—C24	121.3 (2)
Er1—O17—H17A	132.7	C17—C18—C24	118.9 (2)
Er1—O17—H17B	114.6	C20—C19—C18	120.2 (3)
H17A—O17—H17B	112.6	C20—C19—Cl9	120.8 (2)
Er1—O18—H18A	126.5	C18—C19—Cl9	119.0 (2)
Er1—O18—H18B	119.5	C21—C20—C19	120.2 (2)
H18A—O18—H18B	113.5	C21—C20—Cl10	120.2 (2)
Er1—O19—H19A	111.5	C19—C20—Cl10	119.6 (2)
Er1—O19—H19B	135.1	C20—C21—C22	119.9 (2)
H19A—O19—H19B	113.3	C20—C21—Cl11	120.4 (2)
H20A—O20—H20B	110.8	C22—C21—Cl11	119.7 (2)
C2—C1—C6	119.6 (2)	C17—C22—C21	120.1 (3)
C2—C1—C7	118.1 (2)	C17—C22—Cl12	120.4 (2)
C6—C1—C7	122.2 (2)	C21—C22—Cl12	119.4 (2)
C1—C2—C3	120.5 (2)	O9—C23—O10	125.1 (3)
C1—C2—C8	118.6 (2)	O9—C23—C17	123.2 (2)
C3—C2—C8	120.9 (2)	O10—C23—C17	111.7 (2)
C2—C3—C4	119.7 (2)	O11—C24—O12	125.2 (3)
C2—C3—Cl1	120.2 (2)	O11—C24—C18	120.9 (2)
C4—C3—Cl1	120.1 (2)	O12—C24—C18	113.9 (2)
			
O14—Er1—O1—C7	−21.9 (2)	C11—C12—C13—C14	2.5 (4)
O15—Er1—O1—C7	65.3 (2)	Cl6—C12—C13—C14	−176.5 (2)
O18—Er1—O1—C7	129.8 (2)	C11—C12—C13—Cl7	−177.5 (2)
O17—Er1—O1—C7	−171.3 (2)	Cl6—C12—C13—Cl7	3.6 (3)
O13—Er1—O1—C7	38.4 (3)	C10—C9—C14—C13	−2.9 (4)
O19—Er1—O1—C7	−132.6 (2)	C15—C9—C14—C13	177.9 (2)
O16—Er1—O1—C7	−92.5 (2)	C10—C9—C14—Cl8	177.0 (2)
C6—C1—C2—C3	1.6 (4)	C15—C9—C14—Cl8	−2.1 (3)
C7—C1—C2—C3	178.4 (2)	C12—C13—C14—C9	0.4 (4)
C6—C1—C2—C8	179.1 (2)	Cl7—C13—C14—C9	−179.7 (2)
C7—C1—C2—C8	−4.1 (3)	C12—C13—C14—Cl8	−179.6 (2)
C1—C2—C3—C4	0.0 (4)	Cl7—C13—C14—Cl8	0.4 (3)
C8—C2—C3—C4	−177.4 (3)	C14—C9—C15—O5	−74.5 (3)
C1—C2—C3—Cl1	178.8 (2)	C10—C9—C15—O5	106.4 (3)
C8—C2—C3—Cl1	1.4 (3)	C14—C9—C15—O6	106.8 (3)
C2—C3—C4—C5	−1.8 (4)	C10—C9—C15—O6	−72.4 (3)
Cl1—C3—C4—C5	179.4 (2)	C11—C10—C16—O8	−59.8 (4)
C2—C3—C4—Cl2	178.7 (2)	C9—C10—C16—O8	117.9 (3)
Cl1—C3—C4—Cl2	−0.1 (3)	C11—C10—C16—O7	120.8 (3)
C3—C4—C5—C6	1.9 (4)	C9—C10—C16—O7	−61.5 (3)
Cl2—C4—C5—C6	−178.6 (2)	C22—C17—C18—C19	0.9 (4)
C3—C4—C5—Cl3	−177.9 (2)	C23—C17—C18—C19	−179.1 (2)
Cl2—C4—C5—Cl3	1.6 (4)	C22—C17—C18—C24	178.8 (2)
C2—C1—C6—C5	−1.5 (4)	C23—C17—C18—C24	−1.1 (4)
C7—C1—C6—C5	−178.2 (2)	C17—C18—C19—C20	−0.6 (4)
C2—C1—C6—Cl4	179.67 (19)	C24—C18—C19—C20	−178.4 (2)
C7—C1—C6—Cl4	3.0 (4)	C17—C18—C19—Cl9	177.91 (19)
C4—C5—C6—C1	−0.3 (4)	C24—C18—C19—Cl9	0.0 (3)
Cl3—C5—C6—C1	179.6 (2)	C18—C19—C20—C21	−0.3 (4)
C4—C5—C6—Cl4	178.6 (2)	Cl9—C19—C20—C21	−178.73 (19)
Cl3—C5—C6—Cl4	−1.6 (3)	C18—C19—C20—Cl10	179.69 (19)
Er1—O1—C7—O2	6.4 (4)	Cl9—C19—C20—Cl10	1.3 (3)
Er1—O1—C7—C1	−170.47 (16)	C19—C20—C21—C22	0.8 (4)
C2—C1—C7—O2	−61.0 (3)	Cl10—C20—C21—C22	−179.20 (19)
C6—C1—C7—O2	115.7 (3)	C19—C20—C21—Cl11	−178.69 (19)
C2—C1—C7—O1	116.2 (3)	Cl10—C20—C21—Cl11	1.3 (3)
C6—C1—C7—O1	−67.1 (3)	C18—C17—C22—C21	−0.4 (4)
C1—C2—C8—O3	−63.3 (3)	C23—C17—C22—C21	179.6 (2)
C3—C2—C8—O3	114.1 (3)	C18—C17—C22—Cl12	177.55 (19)
C1—C2—C8—O4	116.1 (3)	C23—C17—C22—Cl12	−2.5 (3)
C3—C2—C8—O4	−66.4 (3)	C20—C21—C22—C17	−0.4 (4)
C14—C9—C10—C11	2.6 (4)	Cl11—C21—C22—C17	179.05 (19)
C15—C9—C10—C11	−178.2 (2)	C20—C21—C22—Cl12	−178.41 (19)
C14—C9—C10—C16	−175.2 (2)	Cl11—C21—C22—Cl12	1.1 (3)
C15—C9—C10—C16	4.0 (4)	C22—C17—C23—O9	−55.0 (4)
C9—C10—C11—C12	0.3 (4)	C18—C17—C23—O9	125.0 (3)
C16—C10—C11—C12	178.0 (2)	C22—C17—C23—O10	125.6 (3)
C9—C10—C11—Cl5	179.8 (2)	C18—C17—C23—O10	−54.4 (3)
C16—C10—C11—Cl5	−2.5 (4)	C19—C18—C24—O11	105.4 (3)
C10—C11—C12—C13	−2.8 (4)	C17—C18—C24—O11	−72.5 (4)
Cl5—C11—C12—C13	177.7 (2)	C19—C18—C24—O12	−75.8 (3)
C10—C11—C12—Cl6	176.1 (2)	C17—C18—C24—O12	106.3 (3)
Cl5—C11—C12—Cl6	−3.4 (3)		

### Hydrogen-bond geometry (Å, º)

**Table:**

*D*—H···*A*	*D*—H	H···*A*	*D*···*A*	*D*—H···*A*
O7—H7···O6^i^	0.82 (2)	1.77 (2)	2.566 (3)	162 (3)
O10—H10···O4^ii^	0.82 (3)	1.77 (3)	2.566 (3)	164 (3)
O12—H12···O5^iii^	0.82 (3)	1.77 (2)	2.583 (3)	174 (5)
O13—H13*A*···O9^iv^	0.84	2.57	2.969 (3)	110
O13—H13*A*···O2^v^	0.84	2.03	2.814 (3)	155
O13—H13*B*···O20^vi^	0.84	1.93	2.733 (3)	159
O14—H14*A*···O2^v^	0.85	1.82	2.663 (3)	169
O14—H14*B*···O3^vii^	0.85	1.92	2.724 (3)	157
O15—H15*A*···O3	0.84	2.03	2.868 (3)	169
O15—H15*B*···O4^viii^	0.85	1.90	2.738 (3)	168
O16—H16*A*···O8^ix^	0.85	1.95	2.774 (3)	166
O16—H16*B*···O3^vii^	0.85	1.98	2.764 (3)	153
O17—H17*A*···O6^ix^	0.84	1.92	2.750 (3)	168
O17—H17*B*···Cl5^ii^	0.84	2.83	3.614 (3)	157
O18—H18*A*···O20^vi^	0.84	1.93	2.741 (4)	161
O18—H18*B*···O5^x^	0.84	2.30	2.809 (4)	119
O19—H19*A*···O11^iv^	0.84	2.09	2.909 (3)	167
O19—H19*B*···O5^xi^	0.84	2.51	3.119 (3)	131
O19—H19*B*···O8^ix^	0.84	2.31	3.010 (3)	141
O20—H20*A*···O11^vii^	0.85	2.04	2.781 (3)	146
O20—H20*B*···O10	0.85	2.17	2.834 (3)	134
O20—H20*B*···O12	0.85	2.59	3.080 (3)	118

Symmetry codes: (i) −*x*+1, −*y*+2, −*z*; (ii) −*x*+1, −*y*+1, −*z*+1; (iii) *x*, *y*−1, *z*; (iv) *x*+1, *y*, *z*+1; (v) −*x*+2, −*y*+1, −*z*+2; (vi) *x*, *y*, *z*+1; (vii) *x*+1, *y*, *z*; (viii) −*x*+1, −*y*+1, −*z*+2; (ix) −*x*+2, −*y*+1, −*z*+1; (x) *x*, *y*−1, *z*+1; (xi) *x*+1, *y*−1, *z*+1.
